# A functional chitosan-based hydrogel as a wound dressing and drug delivery system in the treatment of wound healing

**DOI:** 10.1039/c7ra13510f

**Published:** 2018-02-16

**Authors:** He Liu, Chenyu Wang, Chen Li, Yanguo Qin, Zhonghan Wang, Fan Yang, Zuhao Li, Jincheng Wang

**Affiliations:** Orthopaedic Medical Center, The Second Hospital of Jilin University Changchun 130041 P. R. China heliu@ciac.ac.cn cathwang0111@hotmail.com evanlee1357@163.com qinyanguo@hotmail.com wangzhjlu@outlook.com 531040439@qq.com lizuhao1992@163.com jinchengwang@hotmail.com; Hallym University 1Hallymdaehak-gil Chuncheon Gangwon-do 200-702 Korea

## Abstract

Functional active wound dressings are expected to provide a moist wound environment, offer protection from secondary infections, remove wound exudate and accelerate tissue regeneration, as well as to improve the efficiency of wound healing. Chitosan-based hydrogels are considered as ideal materials for enhancing wound healing owing to their biodegradable, biocompatible, non-toxic, antimicrobial, biologically adhesive, biological activity and hemostatic effects. Chitosan-based hydrogels have been demonstrated to promote wound healing at different wound healing stages, and also can alleviate the factors against wound healing (such as excessive inflammatory and chronic wound infection). The unique biological properties of a chitosan-based hydrogel enable it to serve as both a wound dressing and as a drug delivery system (DDS) to deliver antibacterial agents, growth factors, stem cells and so on, which could further accelerate wound healing. For various kinds of wounds, chitosan-based hydrogels are able to promote the effectiveness of wound healing by modifying or combining with other polymers, and carrying different types of active substances. In this review, we will take a close look at the application of chitosan-based hydrogels in wound dressings and DDS to enhance wound healing.

## Abstract 2

Functional active wound dressings are expected to provide a moist wound environment, offer protection from secondary infections, remove wound exudate and accelerate tissue regeneration, as well as to improve the efficiency of wound healing.

## Graphical abstract

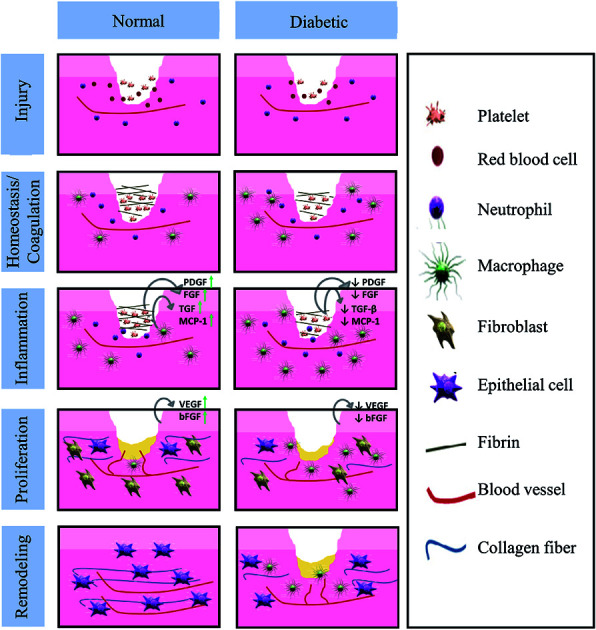

## 1. Introduction

**Fig. 1:**
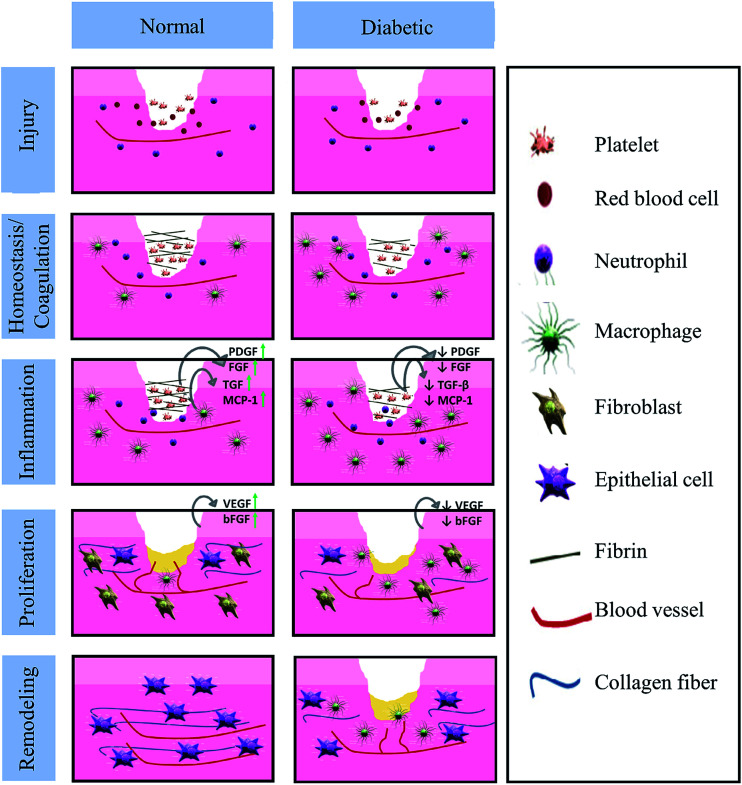
Differences in the normal and diabetic wound healing phases (Reprint with permission from L. I. F. Moura *et al.*^5^).

As the largest human organ, skin reaches 10% of the total body mass, and acts as a protective barrier against the environment.^1^ Besides this physical protective function, skin is also responsible for sensory detection, thermoregulation, fluid homeostasis and immune surveillance.^2^ Normally, the human body is able to restore skin integrity after injury with a minimal scar *via* a complex and interactive process. The various processes of acute tissue repair are divided into a sequence of four time-dependent phases: coagulation and hemostasis, inflammation, proliferation and remodeling.^3^ The normal and chronic wound (such as in diabetes) healing processes are presented in Fig. 1.^4,5^ However, the healing process could be interrupted by a series of factors, such as local factors (oxygenation, wound infection, foreign body, venous sufficiency, wound area, depth, local tension and pressure) and systemic factors (age and gender, sex hormones, stress, ischemia, diseases, obesity, medications, alcoholism, smoking, immunocompromised conditions and nutrition).^6–9^ As a series of factors affect wound healing, medical treatment is necessary.^10^ In fact, the number of diseases resulted from wounds related to infection has increased in the past few years. Therefore, a wide range of wound care products have been developed to improve the life quality of those who suffer from wounds.

Before the 1960s, wound dressings were just considered as so-called passive products with a minimal role in healing process. The pioneering research of Winter *et al.* initiated the concept of an active involvement of a wound dressing in establishing and maintaining an optimal environment for wound repair.^11^ This awareness resulted in the development of wound dressings from traditional passive materials to functional active dressings. Through interacting with the wound where they cover, functional active dressings create and maintain a moist environment for wound healing. Regardless of trauma, burns, diabetic foot or postoperative incision, the application of efficient wound dressings is an important therapeutic method. An ideal wound dressing is expected to provide a moist wound environment, offer protective role in secondary infections, remove wound exudate and promote tissue regeneration, and to improve the quality of wound healing.^5,12^ Taking above factors into consideration, hydrogel has great potential as wound dressings.^13,14^

Hydrogel is made up of a three-dimensional (3D) network of hydrophilic polymers.^15^ The network confers an insoluble behavior to the polymeric system and allows the hydrogels to absorb from 10–20% (an arbitrary lower limit) to up to thousands of times their equivalent weight in water until the process reaches an equilibrium state.^10,16^ They are mainly employed to dry to-moderately draining wounds, to promote autolytic debridement in necrotic wounds and in granulating wounds. Fully swollen hydrogels have a number of common physical properties in living tissues, such as soft, elastic, and low interfacial tension. The elastic properties of the hydrogel can reduce the stimulation of the surrounding tissues. Low interfacial tension between the hydrogel surface and body fluid can decrease the absorption of proteins and cell adhesion to a maximum, thereby ameliorating the chance of a negative immune response.^12,13,17^ Many polymers hydrogels, such as poly(acrylate acid) (PAA), poly(ethylene glycol) (PEG), and poly(vinyl alcohol) (PVA), can increase the retention time of the drug and the permeability of the tissue.^17^ Due to the composition and mechanical aspects, the properties of the hydrogel are similar to the natural extracellular matrix (ECM), so the hydrogel not only serves as the supporting material of the cells in the tissue regeneration process, but also delivers a drug payload.^18,19^

From another aspect, chitin is a natural biological macromolecule polymer and one of the most abundant polysaccharide in nature that exists in some of the shell of crab, shrimp, insects, algae and bacterial cell walls.^20,21^ Extensive sources and low cost make the chitin in the application of biological materials to be valued.^22^ Chitin is insoluble in aqueous solution, so it is usually transformed into chitosan to increase the solubility. The main difference between chitosan and chitin is the content of acetyl group in C-2 position.^23^ Chitosan comprises copolymers of glucosamine and *N*-acetyl-glucosamine units linked by β-1,4-glycosidic linkages.^24^ Chitosan is generally considered to be a biodegradable, biocompatible, non-antigenic, non-toxic, biologically adhesive, antimicrobial, biological activity, with a hemostatic effect.^25–27^ Chitosan and its derivatives have been widely used in the fields of medicine, cosmetics, wound dressings, biochemical separation systems, tissue engineering and some other fields.^28^ These and other positive features, such as hydrophilic and a net cationic charge, make chitosan a suitable polymer for the delivery of other active ingredients like drugs, growth factors, stem cells and peptides.^12^ Different formulations of chitosan-based hydrogel wound dressings can promote wound healing at different periods, and ease the unfavorable factors that affect wound healing. Because of the ability to accelerate wound contraction and healing, chitosan-based hydrogels are regarded as an occlusive dressing for wound healing.^29^ The commercially available wound dressings of chitosan are in the form of non-wovens, hydrogels, films and sponges. A briefly summary of some chitosan-based wound dressing trademarks that are already commercially available are presented in Table 1.^30^

**Table 1:** Some commercial chitin- and chitosan-based wound dressings

Trademarks	Characteristics
Chitipack P® Eisai Co	Chitin-based. Swollen chitin disperse in poly(ethylene terephthalate). Favors early granulation tissue formation. For defects difficult to suture and large skin defects
Chitipack S® Eisai Co	Chitin-based. Sponge-like chitin obtains from squid. Favors early granulation tissue formation, no retroactive scar formation. Suitable for traumatic wounds and surgical tissue defects
Tegasorb® 3M	Chitosan-based. Containing chitosan particles will swell while absorbing exudate and forming a soft gel. A layer of waterproof Tegaderm® film dressing covers the hydrocolloid. Suitable for leg ulcers, sacral wounds, chronic wounds
Chitoflex® HemCon	Chitosan-based. Antibacterial and biocompatible. It combines strongly to tissue surfaces and forms a flexible barrier, which can seal and stabilize the wound. For stuffing into a wound track to control severe bleeding
Chitopack C® Eisai	Chitosan-based. Cotton-like chitosan. Repair body tissue completely, rebuild normal subcutaneous tissue and regenerate skin regularly
Chitopoly® Fuji spinning	Chitosan-based. Chitosan and polynosic Junlon poly(acrylate) for preparing antimicrobial wears. For preventing dermatitis
Chitoseal® Abbott	Chitosan-based. Good biocompatibility and hemostatic function. For bleeding wounds

**Fig. 2:**
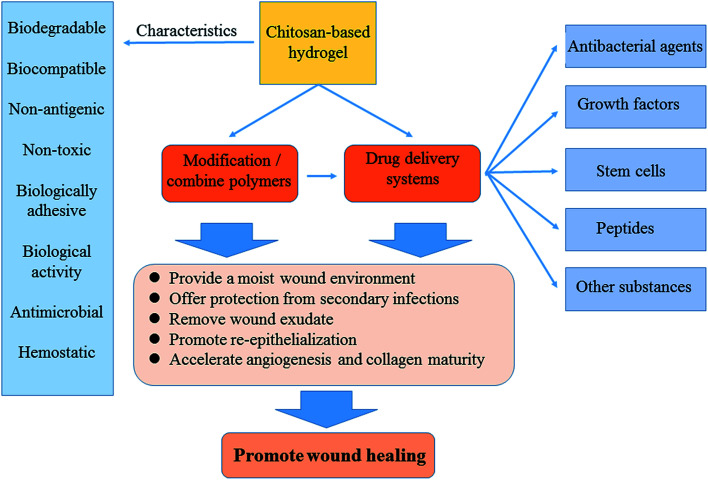
Application of chitosan-based hydrogel dressings. The unique biological properties of chitosan-based hydrogels enable it to serve both as a wound dressing and as a drug delivery system to deliver active substances, which could further promote wound healing.

In this review, we will analyze and summarize the various classes of chitosan-based hydrogels, study their properties and applications, and present recent advances in using natural polysaccharide, chitosan, preparation of hydrogel for wound healing and controlled drug delivery (Fig. 2).

## 2. Chitosan-based hydrogels for wound healing

The characteristics of hydrogels critically depend on the employed polymers and on their interactions within the network. Hydrogels are known as either chemical when their network is covalently cross-linked or physical when the network is sustained by molecular entanglements and/or secondary attractions, including electrostatic interactions, hydrogen bonding or hydrophobic forces. The reversibility of these hydrogels comes from the disruption of the above network interactions *via* modifications in physical conditions such as ionic strength, pH, temperature, stress or specific solutes. Hydrogels can be generated from lots of polymers, and they are classified according to the source of these macromolecules: synthetic, natural or a combination of both.^16,31^

Chitosan is considered as an ideal material for hydrogels due to its biodegradable, biocompatible, non-toxic, antimicrobial, biologically adhesive, biological activity and hemostatic effect, as well as its amino and hydroxyl groups can be easily reacted and chemically modified, thus allowing a high chemical versatility. The conditions employed for amino group chemical modification may interfere with the final degree of deacetylation and therefore with the cationic nature of the obtained materials.^32^ The positively regulatory factors makes chitosan more susceptible to interact with negatively charged molecules such as proteins, anionic polysaccharides and nucleic acids in bacterial membrane, which is the key to antibacterial properties.^33,34^ Chitosan-based materials usually exhibit a positive charge (at typical wound pH values), film-forming capacities, mild gelation characteristics and strong wound tissue adhesive properties.^35^ Chitosan can interact with mucus and epithelial cells, and finally result in opening of cellular tight junctions thus increasing the paracellular permeability of the epithelium. Besides, other structural elements of this polymer are likely to contribute to their penetration-enhancing activity.^36^

Wound healing is a dynamic process involving many molecules and cells, such as mediators, ECM, blood cells and parenchymal cells.^37^ Chitosan-based hydrogels play a positive role in various stages of wound healing. (i) Coagulation and hemostasis, beginning immediately after injury, take place in the wound, which can prevent exsanguination and provide a matrix for invading cells that are needed in the later phases of healing.^38^ Platelets are the most important component in blood coagulation by releasing some cytokines to enhance the healing process.^39^ Chitosan promotes surface-induced thrombosis and blood coagulation and accelerates coagulation *in vivo* by influencing the activation of platelets. Chitosan is a hemostat, which helps in natural blood clotting and blocks nerve endings, thus reducing pain.^40^ (ii) The inflammatory phase of wound healing starts shortly thereafter.^41^ This phase is dominated by inflammatory reactions mediated by cytokines, chemokines, growth factors, and their actions on cellular receptors. Intracellular signaling cascades are activated, contributing to cell proliferation, migration, and differentiation. In addition, chemoattractant factors recruit different cell types, such as granulocytes and macrophages, to the wound site, thus initiating wound repair.^42^ In this process, chitosan-based hydrogels can regulate the activity of related cells and factors releasing, thus forming an appropriate inflammatory microenvironment conducive for healing. Previous studies have shown that chitosan-based dressings can accelerate different tissues repairing and regulate secretion of the inflammatory mediators such as interleukin 8, prostaglandin E, interleukin 1β and others.^29^ Other works also indicated that chitosan-based hydrogels could enhance the inflammatory functions of polymorphonuclear leukocytes, macrophages and neutrophils, promoting tissue granulation to an appropriate inflammatory response.^43^ (iii) Proliferation, which starts from 2 to 10 days after the injury and encompasses the major healing processes, is characterized by proliferation and migration in different types of cells. The proliferative phase includes neoangiogenesis, formation of granulation tissue and ECM, re-epithelialization.^44^ Chitin and chitosan could induce Platelet-Derived Growth Factor (PDGF)-AB and Transforming Growth Factor (TGF)-β1 releasing from the platelets, particularly with a high concentration chitosan.^39^ Chitosan provides a non-protein matrix for 3D tissue growth and activates macrophages for tumoricidal activity. Chitosan will gradually depolymerize to release *N*-acetyl-β-d-glucosamine. As a result, chitosan-based hydrogels could stimulate fibroblast proliferation, angiogenesis, regular collagen deposition and increase level of natural hyaluronic acid (HA) synthesis at the wound site. It helps in faster wound healing and scar prevention.^29,30,45^ (iv) Remodeling: content and arrangement of collagen fibers in scar tissue are adjusted by the action of various enzymes and stress, in order to adapt to physiological work, and results in the development of normal epithelium and maturation of the scar tissue. The *N*-acetyl glucosamine (NAG) present in chitin and chitosan is a major component of dermal tissue which is essential to the repair of scar tissues.^46^ In particular, chitosan films of low deacetylation degree have already proved to be efficient in dressing superficial wounds.^47^

**Fig. 3:**
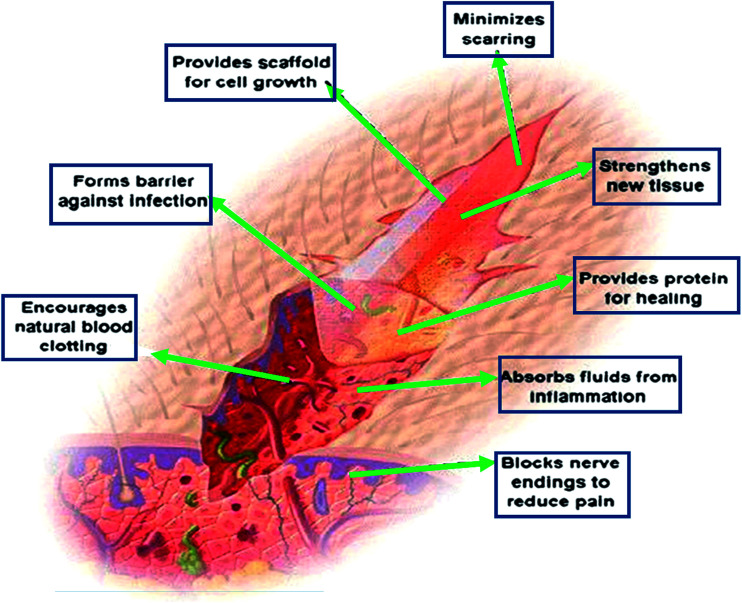
The mechanisms of chitosan-based hydrogels to promote wound healing. Chitosan provides a non-protein matrix for three dimensional tissue growth and activates macrophages for tumoricidal activity. It stimulates cell proliferation and histoarchitectural tissue organization. Chitosan is a hemostat, which helps in natural blood clotting and blocks nerve endings reducing pain (Reprint with permission from R. Jayakumar *et al.*^29^).

Chitosan-based hydrogels can not only promote wound healing at different wound healing stages, but also alleviate the factors against wound healing. For the excessive inflammatory and chronic wound infection, chitosan-based hydrogels have unique advantages. Inflammatory response is the basis of wound healing, but excessive inflammation can lead to necrosis of local tissue cells, which is a factor that hinders wound healing. If it is not timely controlled, it may lead to a systemic infection, which will make the wound healing delayed, and even can be a threat to life. On the other hand, it is easy for bacteria to settle and breed on the chronic wounds, such as diabetic foot ulcer. The presence of infection, bacteria and inflammatory cells increased the consumption of oxygen and other nutrients, fibroblast metabolism were damaged. The release of protease and oxygen free radicals after the neutrophil phagocytic bacteria in the infected area will destroy the tissue, and thus the collagen was dissolved other than deposited. The exudation and the increased local tension make wound dehiscence, which results in delaying wound healing.^48,49^ Chitosan-based hydrogels can exert its advantages on this situation, because of its anti-inflammatory and antibacterial properties, thus provide a suitable microenvironment for healing, inhibiting the inflammatory reaction in the wound and controlling the infection.^33,34^ In addition, if loaded with antimicrobial agents, it can further inhibit microorganisms, thereby accelerating wound healing. As drug delivery system (DDS), chitosan-based hydrogels, which load with active substances (such as growth factors or stem cells), can promote wound healing and it will be discussed as following. Besides the above mechanisms to promote wound healing, chitosan-based hydrogels can also be as a barrier to avoid microorganism proliferation and invasion, and provide scaffold for cell growth, which is shown in Fig. 3.^29^

## 3. Application

As described above, chitosan plays an important role in wound healing, so it is widely used in wound dressings. Here, we will describe its application of wound dressings in two aspects, as wound dressings and DDS. As wound dressings, the physical, chemical and mechanical properties of chitosan can be enhanced by modification, as well as complexed or cross-linked with other polymers and/or cross-linking agents. By this approach, it is possible to design chitosan-based hydrogel dressings with improved healing properties. They include increased exudate absorption capacity, enhanced adherent and anti-bacterial capacity, stimulation of angiogenesis and re-epithelialization of skin tissue and collagen deposition, and sustained delivery of drugs.^50,51^ In addition, as DDS, due to its unique properties, chitosan is a suitable polymer for the delivery of other active ingredients, such as drugs, growth factors, stem cells, peptides and *etc.*, to provide a therapeutic payload that can be more effective in the treatment of local wounds.^52^ The main achievements obtained recently regarding chitosan-based hydrogels as wound dressings or DDS for wound healing will be discussed in the following part.

### 3.1. Chitosan-based hydrogels as wound dressing

In terms of wound healing, chitosan-based hydrogels could provide a moist wound environment, offer protection from secondary infections, remove wound exudate, be biocompatible, induce faster wound healing, and produce smoother scarring. As a result, chitosan-based hydrogels are considered advantageous in their application as a wound dressing material. Especially, when the chitosan is modified and/or combined with other polymers, chitosan-based hydrogel dressings will have some better properties to promote wound healing. In addition, these chitosan-based hydrogels as DDS, such as chitosan–PVA hydrogel,^53^ can deliver bioactive substances (drug, grown factors or stem cells and *etc.*) and controlled release at the wound.

#### 3.1.1. Modified chitosan-based hydrogels

Chitosan could enhance drug absorption due to its mucoadhesive nature. However, the absorption of drugs decrease at higher pH attribute to chitosan's poor solubility at pH greater than 6.0.^54^ Modification of chitosan, through derivatization of the amino and hydroxyl groups by quaternizing with carboxyalkyl, hydroxyalkyl, and acyl derivatives, could increase water solubility at higher pH. As a result, we can improve the biodegradability and biocompatibility, enhance transfection efficiency, and decrease toxicity. Modification has substantially enhanced the biomedical applications of chitosan.^55–57^

There are several common modifications to improve chitosan's properties associated with wound healing. *N*,*N*,*N*-Trimethyl chitosan, *N*-succinyl chitosan, *N*-carboxymethyl chitosan, and thiolated chitosan, have been applied to the preparation of chitosan-based hydrogels.^58^ Carboxymethyl chitosan is water-soluble when pH > 7.^59^ And its antibacterial activity is superior than that of chitosan.^60^*N*-Succinyl chitosan (NSC), formed by the introduction of acyl groups into chitosan, is an amphiprotic derivative containing amine, hydroxyl, and carboxyl groups. The introduction of these groups bestow it with excellent physical, chemical, and biological properties.^58^ NSC has better water retention properties, so it can be exploited in wound dressings. Straccia *et al.* synthesized NSC/sodium alginate hydrogel containing micro-cellulose. The composite had antimicrobial activity against *Escherichia coli* (*E. coli*) and *Staphylococcal aureus* (*S. aureus*), improved swelling degree, stability and water vapor transmission rate. This chitosan-based hydrogel was conducive to maintaining a moist environment in the wound bed to enhance regeneration and epithelialization.^61^ In addition, *N*-succinyl chitosan-based hydrogels were studied *in vivo*. The result showed that they significantly enhanced wound healing and prevented wound infection.^62,63^ More chitosan modified studies were used in the hydrogel for wound healing dressings are listed in Table 2.^58,59,64–66^

**Table 2:** The common chitosan modification methods for wound healing dressings

Modification	Remarks
Carboxymethyl chitosan	Enhanced water solubility. The most fully explored derivative of chitosan; it is an amphoteric polymer, whose solubility depends on pH, when pH > 7 is water-soluble
Alkylation chitosan	Very important as amphiphilic polymers based on polysaccharides. Improve the stability of the interfacial film, cationic surfactant adsorbed on the alkyl chain grafted on chitosan, promotes its solubilization
Trimethyl chitosan ammonium	This cationic derivative, water soluble over all the practical pH range, is obtained by quaternization of chitosan. These polymers show good flocculating and antistatic properties
*N*-Methylene phosphonic chitosans	Having good complexing efficiency for cations such as Ca^2+^, and transition metals (Cu(ii), Zn(ii) *etc.*). The complexation provides corrosion protection for metal surfaces. These derivatives were also modified and grafted with alkyl chains to obtain amphiphilic properties
Carbohydrate branched chitosans	These derivatives are water soluble. Carbohydrates can be grafted on the chitosan backbone at the C-2 position by reductive alkylation, which are important as they are recognized by the corresponding specific lectins and thus could be used for drug targeting
Chitosan-grafted copolymers	When graft with different polymers have different properties. One of the most explored derivatives is PEG-grafted chitosan, which has the advantage of being water soluble, depending on the degree of grafting
Thiolated urea derivatives	Thiourea chitosan increases the antibacterial properties
Sugar derivatives	*N*-Succinyl chitosan (NSC) is an amphiprotic derivative containing amine, hydroxyl, and carboxyl groups, have excellent physical, chemical and biological properties, as required for biomedical applications

#### 3.1.2. Combined with other polymers

In addition to modification, chitosan could also be mixed with other polymers to form a complementary and exert the advantages of each component, thereby enhancing the therapeutic effect of wound dressing.

Natural polymers are classified by obtaining from microbial, animal, and vegetal sources that are usually of a protein or polysaccharide nature. Although these naturally occurring polymers can closely simulate the original cellular environment and ECM, and these biomaterials are known to undergo naturally controlled degradation processes. Their large heterogeneity and batch-to-batch variations upon their isolation from animal or vegetal tissues, as well as the poor stability and mechanical performance are the main limitations for their applications.^25,67^ Other concerns include the relatively high cost (namely of protein-based materials) and the associated risk of the transmission of infectious diseases due to the allogenic or xenogenic origins of the original materials.^68^ Except chemical synthesis and/or processing modifications can overcome some of above disadvantages, blending with other polymeric materials (including natural polymers and synthetic polymers) is another viable alternative.^19^ Application of chitosan-based composite hydrogels will be presented and discussed in the following sections.

##### 3.1.2.1. Natural polymers

###### 3.1.2.1.1. Alginate

Alginate is abundant in nature, which has been widely studied and applied in tissue engineering and drug delivery applications,^68^ due to its high biocompatibility, forming gel easily and rapidly under very mild conditions.^69,70^ However, alginate has low cell adhesiveness because of its poor protein adsorption for the hydrophilic nature.^71^ Therefore, alginates were blended with chitosan to enhance cell interaction, adhesion, and proliferation.^72,73^

Coacervates of alginate and chitosan were prepared to synthesis hydrogel. The dressing promoted the cell proliferation and accelerated the wound closure.^74^ In addition, Sukumar *et al.* reported a new hydrogel containing silk, chitosan, alginate, dextrin, and recombinant human epidermal growth factor (rhEGF). This hydrogel promoted the healing process of deep diabetic wound in rats, and showed advantages in the context of tissue engineering.^75^

Chitosan and alginate incorporated with curcumin and honey (CHS) could be formulated by a simple mixing and situ polymerisation method. The optimised CHS had a good swelling capacity, tensile strength, drug diffusion, bio-adhesion, and water vapour transmission. *In vivo* results indicated that the dressing induced tissue granulation and re-epithelialisation rapidly. The wounds completely healed within one week.^76^ The result was similar to the studies by Dai *et al.*, who reported the wound healing property of non-medicated alginate-chitosan hydrogel.^77^

However, alginate-based hydrogels may present unpredictable and uncontrollable degradation resulting from the loss of divalent cation cross-linkers.^78^ To overcome this issue, covalent/ionic cross-linking with chitosan was employed. Han *et al.* utilized carboxylate moieties on alginate and protonated amines on chitosan to form polyelectrolyte complex (PEC), which exhibited higher mechanical strength and better thermal stability. This method is also used for chitosan and hyaluronic acid (HA), which will be described in the next section.^79^

###### 3.1.2.1.2. HA

HA is a natural polysaccharide, namely a non-sulfated glycosaminoglycan, which is also referred as hyaluronan due to it usually exists *in vivo* as a polyanion but not in the protonated acidic form.^80^ HA presents many importantly physiological functions such as structure and space-filling properties, lubrication, and water sorption and retention abilities.^81^ HA is also an interesting biomaterial for wound dressing since it is known to promote mesenchymal cells and epithelial cells migration and differentiation, thus enhancing collagen deposition and angiogenesis.^80,82,83^ However, hydrogels formed from natural materials are typically mechanically weak that limits their applications.^84,85^ Therefore, it is necessary to produce a material that retains the native conformation of bioactive polymers while improving mechanical properties.

By taking advantage of the poly-anionic nature of HA and the poly-cationic nature of chitosan in aqueous solution, a unique hydrogel material comprised of poly-electrolytic complex (PEC) fibers was produced. It gave the matrix structural integrity and elastic properties without chemical or ultraviolet cross-linking. Each component remained its native and biological relevant state.^86^ Beth *et al.* also used chitosan and HA for preparing PEC. As a result, human mesenchymal stem cells (hMSCs) in the PEC were induced to differentiate and form emergent tissue-like features.^87^ In addition, studies involving the using of chitosan–HA hydrogels in wound healing have been reported. Novel hydrogels, such as HA–poly(vinylphosphonic acid)–chitosan^88^ and aldehyded 1-amino-3,3-diethoxy-propane–HA–chitosan hydrogel^89^ were fabricated and characterized. The results showed these hydrogels enhanced wound healing by promoting cell migration, proliferation, granulation formation, and angiogenesis.

###### 3.1.2.1.3. Cellulose and its derivatives

Cellulose is the primary structural component of plant cell walls and is the most abundant organic polymer on earth. Cellulose-based materials are considered biocompatible due to their reduced inflammatory response for foreign bodies.^90^ Microbial (or bacterial) cellulose, different from plant-origin, is synthesized by various bacteria and has already proved to have great potentials in wound healing applications. Its high mechanical strength, crystallinity, and capacity to retain water mostly arise from its unique nanofibrillar structure.^90,91^

Bacterial nano-cellulose (BNC), of which the biggest feature is fiber diameter, one percent of plant cellulose-only 3–300 nm, is considered to possess incredible potentials in biomedical applications due to its innate unrivaled nano-fibrillar structure and versatile properties.^92^ However, its application is largely restricted by inefficient production and insufficient strength when it is in a highly swollen state. Zhang *et al.* fabricated a fabric skeleton reinforced chitosan/BNC hydrogel, which showed high mechanical reliability and antibacterial activity.^93^ Further *in vivo* study indicated that the wound covered with chitosan/BNC hydrogel was completely filled with new epithelium within 2 weeks, without any significant adverse reactions.^94^

###### 3.1.2.1.4. Collagen and gelatin

Since collagen is one of the major components of human ECMs, and usually considered as an ideal biomaterial for wound dressings. But collagen is difficult to process and hard to control its degradation rate. Gelatin is a collagen derivative, which is usually used to prepare hydrogels for wound dressings.^67^

Collagen/gelatin and chitosan have been widely used to develop scaffolds for skin engineering because of their cell-related signaling properties, such as proliferation, migration and survival. Sanchez *et al.* described the anti-inflammatory activity of chitosan–collagen type I hydrogel, which was permissive for the culture of human adipose-derived mesenchymal stem cells (hADMSC). The results indicated that hADMSC cultured in the hydrogel were viable, proliferative, and can secrete the anti-inflammatory cytokine interleukin-10 (IL-10), and showed good wound repairing potential.^95^ Xiao *et al.* demonstrated that chitosan–collagen hydrogel with immobilized glutamine–histidine–arginine–glutamic acid–aspartic acid–glycine–serine enhanced re-epithelialization and granulation formation, and significantly accelerated diabetic wound closure.^96^ In addition, chitosan scaffold loading with basic fibroblast growth factor (bFGF) contained in gelatin microparticles was studied in chronic ulcers by aged mice. The results suggested this hydrogel was an effective material for growth factor delivery and accelerated healing.^97^ Chitosan–gelatin hydrogels could not only effectively inhibit target microorganisms, but also showed a positive effect on promoting cell proliferation and neovascularization, inducing granulation tissue formation, delivering active substances, and accelerating the wound healing.^98–100^

###### 3.1.2.1.5. Other natural polymers

Many other natural polymers are also incorporated into chitosan-based hydrogels, including fibrin, silk fibroin, dextran, elastin, apigenin, and nerolidol *etc.* Fibrin is a protein produced from fibrinogen. Polymerized fibrin is an important component in the coagulation process, which plays an important role in the wound healing process.^67,101^ Kumar *et al.* developed a chitosan hydrogel/nano-fibrin composite bandages, which enhanced blood clotting, activated platelet activity, and accelerated wound healing.^102^ Chitosan–dextran hydrogel was non-cytotoxic and possessed antimicrobial efficacy, which would be a candidate for wound healing dressings.^103,104^ Chitosan–agarose hydrogel provided an adequate wound healing environment, with high cellular proliferation at hydrogels surface and improved the wound repairing ability.^105,106^ In addition, chitosan mixed with apigenin,^107^ nerolidol^108^ or hemigraphis alternate^109^ also showed the potential for enhancing wound healing.

##### 3.1.2.2. Synthetic polymers

Thanks to the large number of available chemical monomeric entities of potential interest and recent advances in polymer synthesis, many new synthetic biocompatible polymers have been prepared in recent years.^110^ Some of these polymeric materials overcome the problems of natural polymers, because they could be synthesized and processed in a highly controlled way. In addition, some synthetic polymers mainly degrade *via* chemical hydrolysis and are quite insensitive to a number enzymatic processes, hence, their degradation behavior will not vary greatly individually.^111^

Although some studies have shown the potential for using these biomaterials as wound dressings, in each material, individual limitation could be expected.^112^ For wound dressing, naturally derived materials often have desirable biological properties and can influence cell function, but they limited by poor mechanical strengths and fast degradation profiles.^84,85^ In contrast, synthetic polymers provide appropriately 3D environments and have the desired mechanical strengths. However, they lack the bioactive properties of natural material. Therefore, it is necessary to produce hybrid materials by combining synthetic and natural polymers, and retain the desirable characteristics of both materials.

###### 3.1.2.2.1. PVA

Chitosan–PVA hydrogels have been widely used as wound dressing, and a series of studies have shown these composite materials enhanced wound healing as well as antibacterial activities.^53,113–117^ Related research also proved that chitosan–PVA hydrogel exhibited a good bactericidal activity against *E. coli*. The hydrogel with greater chitosan concentration (60% and 80%), had a better cell viability, proliferation, and blood clotting ability.^53,113^ Khodja *et al.* used the chitosan–PVA hydrogel to deal with deep second degree burn rats, and the wound was healed earlier than those treated with paraffin gauze dressing and cotton gauze.^114^ If honey or bee venom was added into the chitosan–PVA hydrogel, it strengthened the anti-inflammatory effect and antibacterial activity, hence, enhanced wound healing.^115–117^ All of these researches indicated chitosan–PVA hydrogels have excellent potential as wound dressings.

###### 3.1.2.2.2. PEG/PEO

PEG is a polyether which is also known as poly(ethylene oxide) (PEO) or poly(oxyethylene) (POE), depending on its molecular weight.^118^ PEG can also be blended with chitosan to improve its inherent solubility, erosion, mechanical and thermal properties, crystallinity, and viscosity.^119^ Chitosan–PEG hydrogel could release drug in a sustained and controlled manner.^120^ Chitosan–PEO hydrogel also could absorb exudate rapidly.^121^ Chen *et al.* indicated the reinforced chitosan–PEG hydrogel has good mechanical property and appropriate degradation rates. Chitosan inhibited inflammatory cells infiltration and enhanced fibroblast proliferation, and PEG promoted epithelial migration. Whether small cuts or full thickness wounds, this reinforced chitosan–PEG hydrogel could promote the wound healing with high quality.^122^

###### 3.1.2.2.3. PVP

Like PVA and PEG, these hydrophilic and biocompatible materials have been extensively studied as wound dressings due to its water absorption and oxygen permeability properties.^5^ Poly(vinyl pyrrolidone) (PVP) is usually blended with other polymers to modify its solubility, delivery property, softness, and elasticity.^123^ Sadiya *et al.* prepared a chitosan–PEG–PVP hydrogel. The water vapour transmission rate was in the range of 2000–3500 g per m^2^ per day, indicating a moderate exudate absorption. When tetracycline hydrochloride was used as model drug within the hydrogel matrix, showed a fast healing with minimum scarring.^124^

###### 3.1.2.2.4. Poly(α-esters)

Polylactide (PLA) is one of aliphatic polyesters, which presents relatively high strength and an appropriate degradation rate with regard to most drug delivery and tissue engineering systems. Besides, PLA provides with good mechanical characteristics, controlled degradability, and excellent biocompatibility. However, its strong hydrophobicity limits the applications.^125^ Chitosan–PLA hydrogel showed a quick absorption capacity, high equilibrate water absorption, and good air permeability, which helped the dressing absorbing excessive exudates, provided a moist environment, and exchanged oxygen in wound healing.^126^ Polyglycolic acid (PGA) is another poly(α-esters) that presents a relatively hydrophilic nature and degrades faster than PLA in aqueous solutions or *in vivo*.^127^ Ching *et al.* presented a novel wound dressing consisting of chitosan and PGA. The hydrogel significantly enhanced the wound healing by suppressing inflammatory phenomena and activating re-epithelialization, besides, easily stripping off from the wound surface without damaging the newly regenerated tissue.^128^

### 3.2. Chitosan-based hydrogels as a drug delivery system

The healing of acute wounds could be accelerated by chitosan alone, however, chronic wounds must heal in a different way. Therefore, the slow release of therapeutic payload may offer a more effective treatment.^12,129^ Despite that a large number of active compounds could serve as therapeutics for wound healing, the inflammatory environment in the wound hinders the drug to enhance healing, and only few candidates have shown clinical effects.^130^ Chitosan-based hydrogels are suitable for the intelligent delivery, which could load with antimicrobial agents, growth factors, stem cells and peptides to balance the biochemical events of inflammation in the chronic wound and enhance healing.

**Fig. 4:**
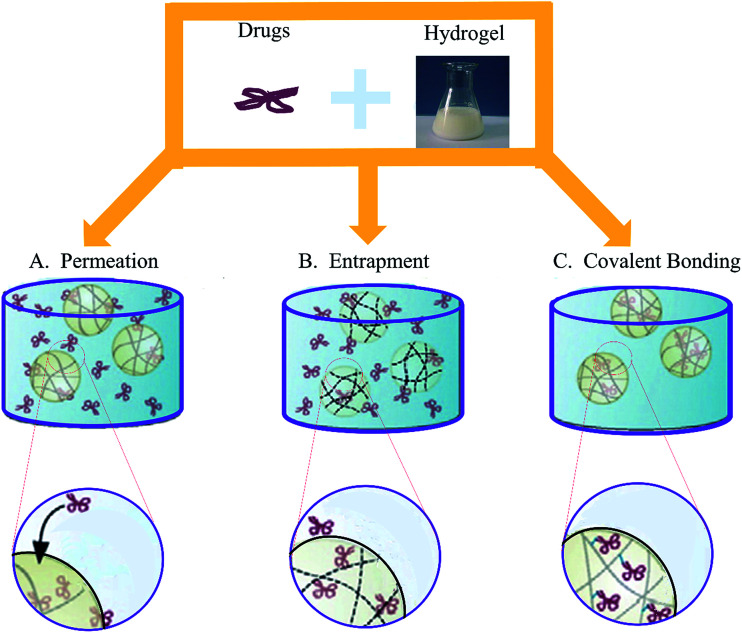
Three main methods of drug loading. (A) The easiest drug loading method is to place the fully formed hydrogel into medium saturated with the therapeutic. (B) In the case of larger drugs and bioligands, the payload must be entrapped during the gelation process. (C) In order to limit the loss of the therapeutic reserve (and the risk of toxic exposure), drugs can be covalently or physically linked to the polymer chains prior to gelation (Reprint with permission from N. Bhattarai *et al.*^12^).

As a DDS, the performance of chitosan-based hydrogels depend not only on the physical and chemical properties of the gel, but also the loading ways between the therapeutic agents and hydrogels. There are three main methods of drug loading: permeation (diffusion), entrapment, and covalent bonding (Fig. 4).^12^ Each method has its own advantages and disadvantages, and should take the hydrogel network and the properties of drugs into consideration when choosing, which are enumerated in Table 3.^33,131–133^ In this section, we will summarize chitosan-based hydrogels as DDS for accelerating wound healing.

**Table 3:** Three different drug loading strategies for chitosan hydrogels

	Permeation	Entrapment	Covalent bonding
Loadable drugs	Small molecules	Small molecules, peptides, proteins, micro/nanospheres	Small molecules, peptides, proteins
Network formations	Physical, covalently cross-linked, and IPN gels	Physical and covalently cross-linked gels	Physical and covalently cross-linked gels
*In situ* gelation possible	NO	YES	YES
Degree of burst release	High	Moderate	None
Smart delivery mechanisms	pH-Sensitive swelling, polymer dissolution and degradation	pH-Sensitive swelling, polymer dissolution and degradation	Enzyme-sensitive release, polymer dissolution and degradation
Release durations	Hours to days	Days and weeks	Days to months
Comments	High loading efficiencies for hydrophilic drugs, low chance of drug deactivation	Suitable for loading hydrophilic and hydrophobic drugs, moderate chance of drug deactivation, chance of toxic material leaching	Best suited for hydrophilic drugs, possible drug deactivation during polymer bonding

#### 3.2.1. Deliver antimicrobial agents

The hydrogel provides a moist environment for wound healing, but the hydrated environment can also facilitate microbial infection, which will prolong or impair the wound healing process.^134^ This is a contradiction, especially in some of the more serious chronic infection wounds. Therefore, hydrogels with antibacterial properties have great potential for clinical application. Chitosan hydrogels itself have antibacterial properties owing to the positively charged amino groups in the chitosan molecule, which could adsorb with negatively charged in bacteria easily.^135^ However, with the increasing of drug-resistant bacteria, chitosan-based hydrogel as a DDS carrying other antimicrobial agents has aroused great concern.

Usually antimicrobial agents are divided into two categories. One is organic antibacterial agents, such as antibiotics, organic mineral salts, and another is inorganic antibacterial agents, such as silver, zinc, copper and metal oxide. Hence, the antimicrobial property of chitosan hydrogel has been developed recently.

##### 3.2.1.1. Organic antibacterial agents

Organic antibacterial agents include a series of substances, such as antibiotics and chemical synthetic drugs, can inhibit and kill bacteria and other microorganisms. They have widely applications in preventing infection, whether oral or injection administration. In recent years, the application of organic antibacterial agent in local wound has attracted the interest of researchers, because it can increase drugs concentration in the wound locally, but not produce a significant antibiotic effect to other parts of the body. It is important to load an antibacterial agent into the dressing, in order to reduce the inflammation caused by bacterial infection during the healing processes, since the wound bed is an ideal environment for microbial growth.^136^

Nimal *et al.* prepared an injectable hydrogel consisting of nanotigecycline and chitosan platelet-rich plasma. Tigecycline was released in a sustained manner and inhibited bacterial growth significantly. This hydrogel was an effective medium for antibiotic delivery and prevented skin infections effectively.^137^ Sadiya *et al.* incorporated tetracycline hydrochloride into chitosan–PEG–PVP hydrogel as an antimicrobial and scar preventive dressing. The composite dressing showed good antimicrobial properties against both type of bacterial strain. Chitosan promoted wound healing with minimum scar and tetracycline hydrochloride provided protection from bacterial invasions.^124^ In addition, chitosan–PVA hydrogel was prepared to delivery minocycline^138^ and gentamycin sulfate,^139^ and chitosan–polyacrylamide (PAM) hydrogel was fabricated to delivery piperacillin–tazobactam.^140^ As well as amikacin,^141^ gentamicin/ciprofloxacin,^142^ ciprofloxacin,^143^ norfloxacin,^144^ sulfadiazine^145^ were loaded into chitosan-based hydrogels to develop the antibacterial function. These studies have proved the efficacy of antibacterial agents contained in chitosan-based hydrogel dressings for decreasing infection, favoring granulation tissue formation, and stimulating faster wound healing.

##### 3.2.1.2. Inorganic antibacterial agents

Drug-resistant bacteria in infected wound is a challenge to wound healing.^146^ Nano metals as inorganic antibacterial agents have good prospect against drug-resistant bacteria with a similar antibacterial mechanism. Nano silver (nAg), for example, could be oxidized on the wound surface when in contact with moisture or wound fluid. Then Ag^+^ ions are released and attached to the bacterial cell membrane. Ag^+^ ions damage the membrane by interacting with sulphur-containing proteins and enter inside the bacteria to disrupt DNA.^147,148^ A large number of studies incorporated the inorganic antimicrobial agents into chitosan-based hydrogels as wound dressings.

###### 3.2.1.2.1. Silver

nAg is a broad spectrum antimicrobial agent *via* multiple mechanisms against microbes, which significantly reduces the chance of developing resistance. nAg has a better effective antimicrobial than ionic silver due to their better permeation and retention effects.^149^ A number of developed wound dressings containing silver (Acticoat™, 3M™ Tegaderm™, Bactigrass®, SilvaSorb®, Fucidin®, PolyMem® Silver) have been approved by Food and Drug Administration.^150,151^

Chitosan-based hydrogel containing silver nanoparticles showed the maximum activity against the resistant bacteria isolating from diabetic foot. This composite prevented the foot infection with multidrug-resistant bacteria, and obviously accelerated wound healing.^152^ In addition, topical formulations based on chitosan/nAg hydrogels have been prepared and their effects on wound healing were studied extensively. nAg were incorporated into nanocomposite chitosan-based hydrogel dressings for full-thickness skin wounds,^153^ bactericidal activity of hydrogel beads based on *N*,*N*,*N*-trimethyl chitosan/alginate complexes loaded with nAg,^154^ antibacterial chitosan/nAg bio-nanocomposite hydrogel beads as DDS,^155^ nAg-containing antimicrobial membrane based on chitosan–tripolyphosphate (TPP) hydrogel for the treatment of wounds.^156^ These results indicated nAg played an important role in antibacterial aspect and had a great application prospect in wound dressing.

The toxicity of nAg can kill microorganisms, but also have the same effect on normal human cells. nAg shows a concentration dependent cytotoxic effect towards human dermal fibroblast cells.^157^ Therefore, establishing a therapeutic window to control nAg within a range can inhibit bacteria but not produce toxicity to human cells, which is the key for the application of chitosan–nAg hydrogel. The chitosan-based hydrogels could release the nAg in a sustained way. At a controllable concentration, silver incorporating into chitosan-based hydrogel show great potential for avoiding infection and enhancing wound healing.

###### 3.2.1.2.2. Zinc

As a necessary element of the human body, zinc is effective on some antibiotic resistant strains due to its complex antibacterial mechanism.^151,158^ Zinc oxide (ZnO) is the main form to exert antibacterial effect. However, there also some studies indicated that the zinc ion also had a significant antibacterial effect, weaker than the silver ion, though.^158,159^

Nair *et al.* reported that ZnO nanoparticles (nZnO) had potent antibacterial activity without adverse effect on normal cells at appropriate concentrations.^160^ To investigate the suitable concentration of zinc playing extensive antibacterial effect with low toxic effects on the cells, a stable DDS was necessary. Kumar *et al.* incorporated nZnO into chitosan hydrogel. The result showed this composite dressing enhanced blood clotting and inhibited bacterial growth without causing toxicity to cells. Furthermore, *in vivo* researches revealed that the nanocomposite promoted re-epithelialization, collagen deposition, and enhanced wound healing. These results indicated this nanocomposite was a potential application for burn wounds, chronic wounds and diabetic foot ulcers.^161^

###### 3.2.1.2.3. Other metals

Titanium dioxide (TiO_2_) nanoparticles have been used in cosmetics and filters, which exhibit potent bactericidal properties and the abilities of eliminate odors.^46^ Slowly release of titanium ions from the nanoparticles can inhibit microbial proliferation, and therefore accelerate wound healing.^162^ The chitosan–TiO_2_ composite membrane had excellent surface properties and bactericidal activities.^163,164^ Studies have indicated that gold (Au) also has a significantly antibacterial activity.^165^ Martins *et al.* successfully prepared *N*,*N*,*N*-trimethyl chitosan/alginate complex-loaded with Au nanoparticles had good biocompatibility and characterized by wound dressing potential.^166^

#### 3.2.2. Deliver growth factors

Growth factors are regulatory peptides synthesized and secreted by fibroblasts, inflammatory cells, endothelial cells, epithelial cells, and platelets. Growth factors can induce cell migration, proliferation, differentiation, and promote the synthesis of ECM.^37,42,167^ Compared with normal wound healing, chronic wound secrets less growth factors in different stages.^5^ In the case of diabetic foot ulcers, a series of multiple mechanisms decrease the peripheral blood flow and local angiogenesis, all of which can hinder wound healing.^168^ Growth factors are divide into several families based on their characteristics. The most relevant growth factor families for wound healing are EGF, FGF, TGF-β, PDGF, and vascular endothelial growth factor (VEGF). The sources and important roles of these growth factors in wound healing are summarized in Table 4.^169–171^

**Table 4:** Major growth factors in wound healing

	Cell sources	Effects during wound healing
EGF	Platelets, macrophages, fibroblasts	Cell motility and proliferation, increased levels in the acute wound, decreased levels in the chronic wound
FGF	Macrophages, endothelial cells, fibroblasts	Angiogenesis and fibroblast mitogen, keratinocyte mitogen and mitogen
TGF-β1, TGF-β2	Platelets, keratinocytes, macrophages, lymphocytes, fibroblasts	Re-epithelialization and inflammation, granulation tissue formation, fibrosis and tensile strength, increased levels in the acute wound, decreased levels in the chronic wound
PDGF	Platelets, keratinocytes, macrophages, endothelial cells, fibroblasts	Chemotaxis, inflammation, granulation tissue formation, matrix remodeling, increased levels in the acute wound, decreased levels in the chronic wound
VEGF	Platelets, neutrophils, macrophages, endothelial cells, fibroblasts	Angiogenesis, granulation tissue formation, increased levels in the acute wound, decreased levels in the chronic wound
IGF	Fibroblasts neutrophils, macrophages, hepatocytes, skeletal muscle	Stimulates wound re-epithelialisation and fibroblast proliferation
HGF	Fibroblasts	Suppression of inflammation, granulation tissue formation, angiogenesis, re-epithelialization

Exogenous growth factors enhancing wound healing were initially promising. However, application of growth factors to the wound directly has several limitations. The half-life is generally short and need repeated administration. They also degrade quickly because of the abundant proteolytic enzymes in the wound environment. Furthermore, sequestration of growth factors by the wound matrix may hinder its binding to receptors at the surfaces of the cells.^172^ Therefore, it is necessary to develop an applicable system to deliver growth factors in order to improve their clinical efficacy. Importantly, chitosan-based hydrogels have unique advantage to become an excellent choice to maximize the effectiveness of growth factors.

EGF incorporated into chitosan–albumin hydrogel microspheres could continuous release more than 3 weeks after subcutaneous implantation in rats.^17^ Pulat *et al.* prepared chitosan–polyacrylamide hydrogel loading with EGF, the composite enhanced fibroblast cells proliferation for longer periods than that of free EGF.^140^ Furthermore, as DDS, sodium carboxymethyl chitosan hydrogel,^172^ chitosan–alginate beads^75^ and Pluronic–chitosan hydrogels^173^ were developed to carry rhEGF and studied in diabetic rats. These results indicated that the chitosan-based hydrogels released rhEGF at the wound sites controllably, enhanced the healing rate, and improved the healing quality significantly.

FGF could stimulate angiogenesis by activating capillary endothelial cells and fibroblasts.^97^ In order to maintain its release stably at the wound site, FGF was incorporated into chitosan-based hydrogels. A series of studies have indicated that aFGF^174^ and bFGF^97,175^ incorporated into chitosan-based hydrogels were effective material for enhancing chronic wounds healing.

Rapid angiogenesis is crucial in skin regeneration, which could promote regeneration, transmit oxygen and nutrients, remove metabolic waste, and decrease the risk of infection.^176^ Incorporated VEGF into the dressing and released in a sustained way could improve angiogenesis and enhance wound healing without signs of reactive or granulomatous inflammatory response.^177^

#### 3.2.3. Deliver stem cells

**Fig. 5:**
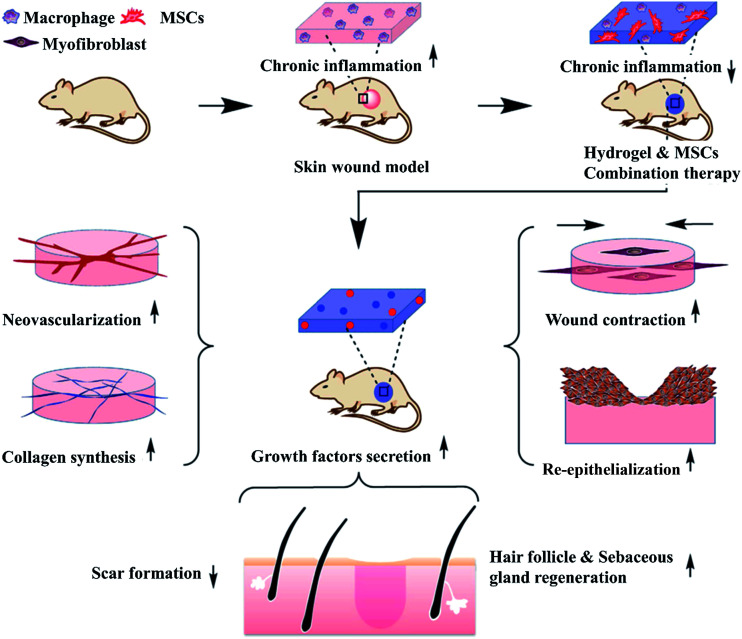
MSC-laden hydrogels can prohibit chronic inflammation and contribute to growth factor secretion, resulting in accelerated wound contraction, ECM secretion, angiogenesis, re-epithelialization, hair follicle and sebaceous gland regeneration and reduced scar formation (Reprint with permission from Chen *et al.*^186^).

In recent years, stem cells in wound repair have become a hot spot attributing to the fact that stem cells can differentiate into epidermal cell phenotypes, up-regulate cytokines and growth factors expression around the wound site. Afterwards, increasing evidence has demonstrated that the paracrine effect of stem cells play the key roles in promoting wound healing.^178–180^ Both bone marrow mesenchymal stem cells (BMSCs) and adipose derived stem cell (ADSCs) have been reported to enhance angiogenesis, promote epithelialization, and affect recruitment or proliferation of macrophages and endothelial progenitor cells during the healing process.^181,182^ The differentiation and growth factors secretion of stem cells are regulated by the microenvironment.^183,184^ However, there is a large amount of cytotoxic and inflammatory mediators in the microenvironment of the wound, which may cause the death of stem cells in the local wound tissue.^185^ The mechanisms of stem cell-laden anti-inflammatory hydrogel enhancing chronic wound healing are described in Fig. 5.^186^ Because chitosan-based hydrogels have the biological advantages of biocompatible, biodegradable, maintaining multipotency of the stem cells, and mimicking ECM, they become excellent delivery systems to protect stem cells in order to maximize the differentiation and paracrine capacity.^187^ Currently, many researchers have focused on stem cells-laden hydrogels to promote wound healing. Compared with alginate hydrogel, stem cells in self-healing chitosan hydrogel proliferated much faster.^188^ Here, we will discuss the application of chitosan-based hydrogels loading with stem cells in wound healing.

BMSCs are reported to enhance wound healing through secreting a series of growth factors^189^ and differentiating into effector cells, thereby accelerating wound closure, vascularization, granulation tissue formation, and re-epithelialization.^190–192^ Considering the aforementioned mechanisms, the active role of BMSCs in wound healing establishes the foundation for their application in treating chronic wound healing. A chitosan/dextran-based injectable hydrogel not only retained BMSCs viability, but also maintained the differentiation capacity and mesenchymal immune-phenotype. In the chitosan/dextran-based hydrogel, BMSCs differentiated into osteocytes and adipocytes successfully. *In vivo* study indicated that the hydrogel was effective prevention of scar formation after surgery.^193^ In addition, hydroxypropyl–chitosan hydrogel^194^ and chitosan–collagen microbeads^187^ were also beneficial to BMSCs adhesion and proliferation, which were suitable materials to deliver BMSCs as wound dressings.

ADSCs have been used in wound healing since they have immune-regulatory and multipotent differentiation capabilities.^195^*In vitro*, chitosan-based hydrogels served as a scaffold could promote ADSCs proliferation and differentiation with low cytotoxicity.^196^ ADSCs-encapsulated chitosan/gelatin hydrogel promoted proliferation of fibroblasts and tube formation of endothelial cells *in vitro*, and promoted wound angiogenesis *in vivo*.^197^ Sanchez *et al.* developed a novel hydrogel with anti-inflammatory activity. Their study showed that the hADSCs cultured in the collagen type I/chitosan/dexamethasone hydrogel were viable, proliferative, and secreted the anti-inflammatory cytokine IL-10 but not the inflammatory cytokine tumor necrosis factor-α. This was for the first time that a native ECM molecule (collagen type I), a biocompatible natural polymer (chitosan), and an inflammation-controlling molecule (dexamethasone) have been combined into a hydrogel that proved to be capable of sustaining mesenchymal stem cells culture.^95^ Chang *et al.* reported an injectable chitosan–HA hydrogel delivered ADSCs significantly accelerated wound closure. The composite hydrogel increased cell proliferation and promoted keratinocyte migration, up-regulated mRNA expressions of VEGF, chemotactic factors and ECM-remodeling matrix metaloproteinases.^198^

In addition, synovial mesenchymal stem cells loaded into hydroxyapatite–chitosan hydrogels. *In vivo* results indicated that the composite hydrogel significantly promoted re-epithelialization, angiogenesis, and collagen maturity around diabetic chronic wound surface.^199^

In general, chitosan-based hydrogels show great promise as stem cells delivery vehicles for tissue regeneration. BMSCs have a wide range of applications, and many studies have demonstrated its effectiveness and safety. Compared with other stem cells, BMSCs have the advantage in terms of healing rate and blood flow of the limbs for ulcer patients.^200^ In comparison to BMSCs, ADSCs acquired *via* liposuction are much easier to access.^201^ In addition, the isolated cells can be cryopreserved while maintaining all their properties intact for up to 6 months, which provides a good potential for ADSCs to become an off-the-shelf product.^202^ This convinced us that chitosan-based hydrogels loaded stem cells to promote wound healing is very promising.

#### 3.2.4. Deliver peptides

Although stem cells and growth factors intend to improve angiogenesis and re-epithelialization, cost and safety issues remain in their applications. Peptides show similar effects with growth factors, but have lower cost and controllable properties.^203,204^ Similarly, a stable delivery system allows peptides to better promote wound healing. Chen *et al.* reported a biomimetic fragment of the laminin mimetic peptide, Ser–Ile–Lys–Val–Ala–Val-conjugated chitosan hydrogel. This composite material significantly promoted BMSCs adhesion and proliferation *in vitro*, and accelerated wound contraction *in vivo*. These results suggested that the peptide-modified chitosan hydrogel significantly improved the function of chitosan in angiogenesis and re-epithelialization of skin.^205^ Xiao *et al.* presented that chitosan–collagen hydrogel with immobilized glutamine-histidine–arginine–glutamic acid–aspartic acid–glycine–serine (an integrin-binding prosurvival peptide derived from angiopoietin-1), treated the full-thickness excisional wounds in a diabetic mice. This composite hydrogel significantly enhanced wound closure *via* faster re-epithelialization and granulation tissue formation.^206^

#### 3.2.5. Deliver other drugs

In addition to the above categories, chitosan-based hydrogels can also deliver some other drugs, such as anti-inflammatory drugs, antioxidants, amino acids, vitamins, and nutrients, which can reduce the inflammatory reaction, well-nourished wound tissue, and promote wound healing.^207^

The inflammatory phase starts within a few minutes of injury up to 24 hours and lasts for about 3 days. This therefore necessitates effective analgesic delivery during this inflammatory period.^208^ Chitosan–PVA hydrogel containing bee venom was developed, and exhibited anti-inflammatory effect, which could be comparable to that of diclofenac gel, a standard anti-inflammatory drug. Combination of chitosan and bee venom significantly accelerated wound healing in diabetic rats.^116^ In addition, ibuprofen,^207^ betamethasone sodium phosphate, streptomycin, and diclofenac^208^ also have the potential as anti-inflammatory drugs delivered in chitosan-based hydrogels. These evidenced chitosan-based hydrogels have significantly potential to control the delivery of anti-inflammatory drugs over a period compatible with the wound healing progresses.

Antioxidants, such as nitric oxide, horseradish peroxidase, and hydrogen peroxide were also studied in chitosan-based hydrogels, which showed a stronger antibacterial activity, stimulated fibroblast proliferation and collagen production, exhibited fast contraction of incision, and accelerated epithelialization and wound healing eventually.^209,210^ In addition, Wu *et al.* added vitamin C into chitosan–PVA hydrogel. Sustained release of the vitamin provided a new system to enhance wound healing in dermal tissue.^211^

## 4. Conclusion

Chitosan-based hydrogel is considered as an ideal material due to its biodegradable, biocompatible, antimicrobial effects, and these properties of chitosan-based hydrogels could be modified by various natural or synthetic polymers. Relative to acute wound, the chronic and complex wounds need to be treated with functional wound dressing, possessing the capacity of releasing therapeutic drugs or growth factors to offer a more effective treatment. To address this, chitosan-based hydrogels have been developed as wound dressings, which can deliver antibacterial agents, growth factors, stem cells, peptides and other active substances in a sustained release manner. The local intervention can solve the problem of systemic toxicity and maintain the effective concentration of the active material in the wound to promote the chronic wounds healing. We believe that as wound dressing and DDS, chitosan-based hydrogels have great potential clinical application in wound healing.

## Conflicts of interest

There are no conflicts to declare.

## Supplementary Material
